# Optimization of human mesenchymal stem cell manufacturing: the effects of animal/xeno-free media

**DOI:** 10.1038/srep16570

**Published:** 2015-11-13

**Authors:** Angelos Oikonomopoulos, Welmoed K. van Deen, Aida-Rae Manansala, Precious N. Lacey, Tamera A. Tomakili, Alyssa Ziman, Daniel W. Hommes

**Affiliations:** 1 Center for Inflammatory Bowel Diseases, Melvin and Bren Simon Digestive Diseases Center, David Geffen School of Medicine, UCLA, Los Angeles, California; 2 Department of Gastroenterology and Hepatology, Leiden University Medical Center, Leiden, The Netherlands; 3 Division of Transfusion Medicine, Department of Pathology and Laboratory Medicine, David Geffen School of Medicine, UCLA, Los Angeles, California.

## Abstract

Due to their immunosuppressive properties, mesenchymal stem cells (MSC) have been evaluated for the treatment of immunological diseases. However, the animal-derived growth supplements utilized for MSC manufacturing may lead to clinical complications. Characterization of alternative media formulations is imperative for MSC therapeutic application. Human BMMSC and AdMSC were expanded in media supplemented with either human platelet lysates (HPL), serum-free media/xeno-free FDA-approved culture medium (SFM/XF), or fetal bovine serum (FBS) and the effects on their properties were investigated. The immunophenotype of resting and IFN-γ primed BMMSC and AdMSC remained unaltered in all media. Both HPL and SFM/XF increased the proliferation of BMMSC and AdMSC. Expansion of BMMSC and AdMSC in HPL increased their differentiation, compared to SFM/XF and FBS. Resting BMMSC and AdMSC, expanded in FBS or SFM/XF, demonstrated potent immunosuppressive properties in both non-primed and IFN-γ primed conditions, whereas HPL-expanded MSC exhibited diminished immunosuppressive properties. Finally, IFN-γ primed BMMSC and AdMSC expanded in SFM/XF and HPL expressed attenuated levels of IDO-1 compared to FBS. Herein, we provide strong evidence supporting the use of the FDA-approved SFM/XF medium, in contrast to the HPL medium, for the expansion of MSC towards therapeutic applications.

Stem cell therapy utilizing mesenchymal stem cells (MSC) has emerged as an alternative approach in various pathologic conditions. MSC have been applied toward the treatment of bone diseases, cartilage repair, myocardial infarction, and auto-immune diseases such as graft versus host disease (GVHD) and inflammatory bowel disease (IBD)^1^,^2^,^3^,^4^,^5^,^6^,^7^,^8^. MSC are self-renewing and multipotent adult stem cells. They are typically obtained from the bone marrow, but can also be isolated from other tissues such as subcutaneous fat, skeletal muscle, amniotic fluid, and placenta among others. More recently, MSC were shown to possess potent immunomodulatory properties and the ability to alter the function of immune cells^9^,^10^,^11^,^12^,^13^. The immunosuppressive properties of MSC can be augmented by pre-stimulation (priming) with pro-inflammatory cytokines such as interferon-γ (IFN-γ)^14^,^15^,^16^,^17^. Recent data from our laboratory have confirmed the immunomodulatory efficacy of IFN-γ primed bone marrow derived MSC (BMMSC) compared to non-primed BMMSC in experimental models of colitis in mice^14^.

Salutary application of MSC requires their *ex vivo* expansion in order to reach appropriate cell numbers to achieve therapeutic outcomes. Thus, identification of optimal culture conditions is a prerequisite for MSC clinical applications. Animal derived growth supplements, such as fetal bovine serum (FBS), have been predominantly used for MSC expansion in clinical protocols. However, utilization of animal derived products bears critical limitations and safety concerns^18^, such as animal derived (xeno) antigens and infectious agents present in FBS that might be transmitted to the recipient of MSC therapy^19^,^20^,^21^,^22^,^23^,^24^,^25^. Moreover, the precise composition of FBS remains unclear and often there are significant variations between lots^26^. To avoid undesirable complications, alternative animal product-free media formulations have been evaluated. Recent efforts have focused on the development of animal serum-free culture media with the utilization of human derived growth supplements for MSC expansion, such as human platelet lysates (HPL)^27^,^28^,^29^,^30^,^31^,^32^,^33^,^34^,^35^,^36^,^37^,^38^,^39^,^40^,^41^. Additionally, chemically-predefined animal and human serum-free culture media have been developed for the expansion of MSC towards clinical applications^42^,^43^,^44^,^45^,^46^. Recently, the Federal Drug Administration (FDA) approved the first commercially available pre-defined xeno-free culture medium specially formulated for the expansion of human MSC^47^.

In order to determine if animal-free/xeno-free media can be used for optimal MSC expansion, we analyzed the functional properties of non-primed and IFN-γ primed BMMSC and adipose derived MSC (AdMSC) expanded in xeno-free media formulations. We found that the pre-defined serum-free media/xeno-free culture medium (SFM/XF) in contrast to HPL-supplemented medium represents an alternative way to expand BMMSC and AdMSC that preserves their functional properties.

## Results

### Effects of alternative culture conditions on the morphology of rested and primed BMMSC and AdMSC

**Figure 1:**
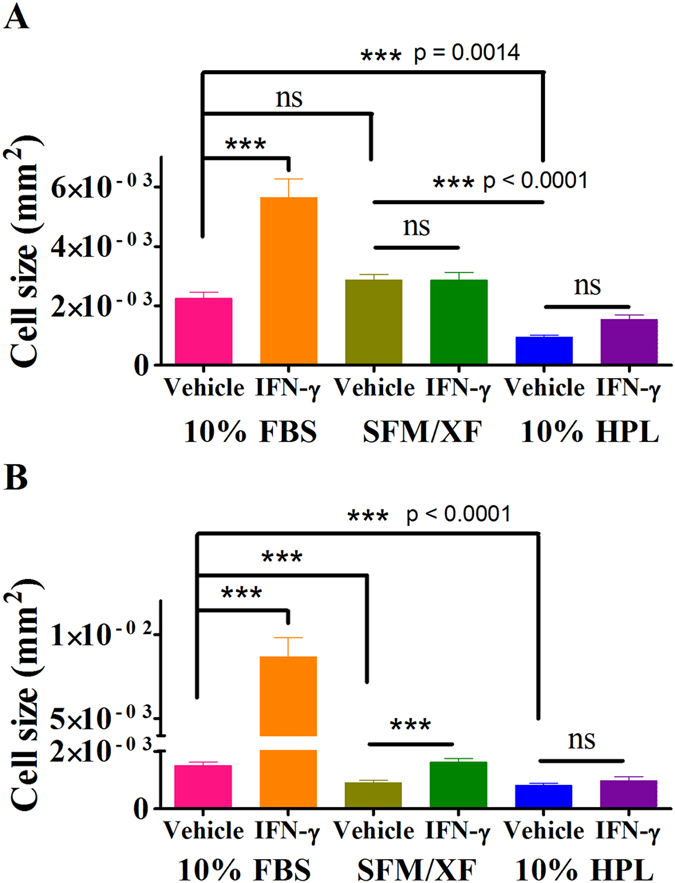
Cell size quantification of resting and IFN-γ primed BMMSC expanded in alternative media formulations. Diagram shows quantitative analysis of (**A**) BMMSC and (**B**) AdMSC surface area in all media formulations 3 days post priming. Data are mean values ± standard error of the mean (s.e.m.).

Resting (vehicle-treated, non-primed) BMMSC cultured in 10%HPL (BMMSC-10%HPL) exhibited an increase in length and decrease in size (area) compared to SFM/XF and FBS treated BMMSC (BMMSC-SFM/XF (p < 0.0001) and BMMSC-10%FBS (p < 0.0014), respectively) (Fig. 1A and Supplementary Figures 1E, C, and A, respectively). No difference in size and shape was observed between BMMSC-SFM/XF and BMMSC-10%FBS under resting conditions (Fig. 1A and Supplementary Figures 1C and A). Similarly, AdMSC-SFM/XF and AdMSC-10%HPL appeared smaller (p < 0.0001) in size compared to AdMSC-10%FBS (Fig. 1B and Supplementary Figures 2C, E, and A, respectively, and Supplementary Tables 1 and 2).

IFN-γ priming increased the cell size (p < 0.0001) and changed the morphology of BMMSC to a less elongated shape in 10%FBS but not in SFM/XF or 10%HPL, compared to the respective resting cells (Fig. 1A and Supplementary Figures 1B, D, and F). Similarly, IFN-γ priming increased (p < 0.0001) the cell size and changed the morphology of AdMSC to a less elongated shape in 10%FBS and SFM/XF but not in 10%HPL (Fig. 1B and Supplementary Figures 2B, D, and F, and Supplementary Tables 1 and 2). The above results show that alternative culture conditions in combination with IFN-γ priming lead to morphological changes in BMMSC and AdMSC. These changes in size and morphology could indicate changes in cell proliferation and/or differentiation; therefore we examined the effects of alternative media formulations on MSC homeostasis.

### Effects of alternative media formulations on the immunophenotypic profile of BMMSC and AdMSC

**Figure 2:**
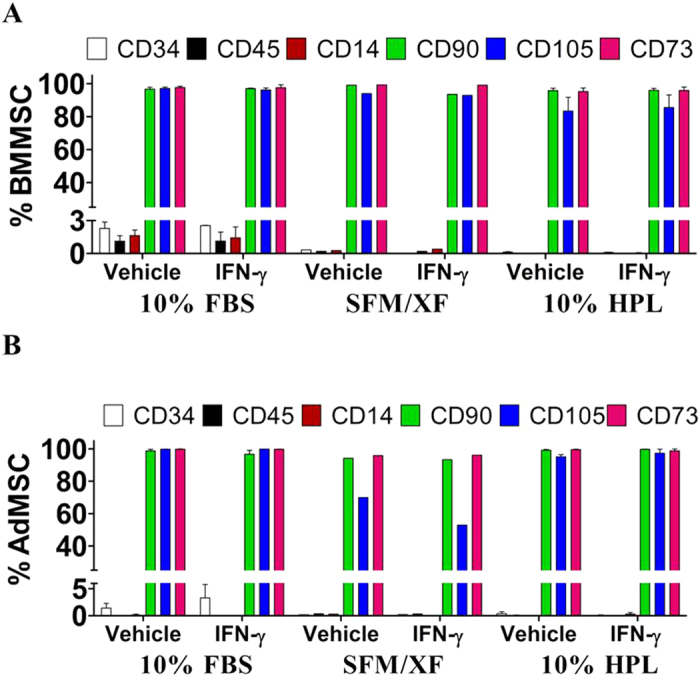
Alternative media formulations do not alter the immunophenotypic profile of resting (non-primed) and IFN-γ primed BMMSC and AdMSC. Diagrams demonstrate the fraction of BMMSC (**A**) and AdMSC (**B**) expressing CD34, CD14, CD45, CD90, CD105 and CD73 cell surface markers in all culture conditions. All data points are generated by performing at least two independent experiments in triplicates. Data are mean values ± s.e.m.

To monitor the identity and the phenotype of BMMSC and AdMSC in alternative media formulations, we examined the expression of six well established cell surface markers (CD34, CD45, CD14, CD90, CD105, and CD73) as well as HLA-DR^48^. BMMSC and AdMSC were negative for the expression of the hematopoietic markers CD14, CD34, and CD45 in all media formulations and positive for the expression of CD90, CD105, and CD73 (Fig. 2A,B). The immunophenotypic profile of both BMMSC and AdMSC was not altered by IFN-γ priming (Fig. 2A,B and Supplementary Tables 1 and 2). Interestingly, in AdMSC-SFM/XF the expression level of CD105 was reduced following IFN-γ priming (Fig. 2B).

We and others have previously shown that HLA-DR is not expressed in resting BMMSC and AdMSC, and it is highly up-regulated following IFN-γ priming^14^. Indeed, resting BMMSC expressed only marginal levels of HLA-DR in all media formulations (Supplementary Figures 3A,C,E,G), while IFN-γ priming resulted in significant up-regulation (p < 0.0001) of HLA-DR in BMMSC-10%FBS, and to a lesser extent, in BMMSC-SFM/XF and BMMSC-10%HPL (p < 0.001) (Supplementary Fig. 3B,D,F,G, and Supplementary Table 1 and Table 2). HLA-DR was highly expressed (p < 0.0001) in AdMSC following IFN-γ priming in all media formulations (Supplementary Figures 4B,D,F,G), compared to HLA-DR-negative resting AdMSC (Supplementary Figures 4A,C,E,G, and Supplementary Tables 1 and 2). The above data shows that alternative culture conditions marginally affect the induction of HLA-DR and potentially the priming efficiency of BMMSC and AdMSC.

### Effects of alternative media formulations on the differentiation capacity of BMMSC and AdMSC

BMMSC and AdMSC retained their adipogenic and osteogenic differentiation capacity in all media formulations as evidenced by immunostaining for lipid droplets (oil Red staining) and alkaline phosphatase (ALP) respectively (Supplementary Fig. 5 and 6). To quantify the extent of adipogenic differentiation in BMMSC and AdMSC we measured the expression levels of adiponectin, and PPAR-γ in both MSC types expanded in all media formulations. Culture in 10%HPL supplemented medium demonstrated the highest levels of adiponectin and PPAR-γ compared to 10%FBS and SFM/XF for both BMMSC (p < 0.0001) and AdMSC (p < 0.04 and p < 0.003, respectively) (Supplementary Figure 7A, B and C, D respectively, and Supplementary Tables 1 and 2).

Next we measured the expression levels of ALP a marker of osteogenic differentiation. BMMSC-10%HPL expressed the highest (p < 0.001) levels of ALP, followed by BMMSC-SFM/XF and BMMSC-10%FBS (Supplementary Figure 8A). Likewise, AdMSC-10%HPL expressed the highest levels of ALP, followed by AdMSC-10%FBS (p < 0.05) and AdMSC-SFM/XF (p < 0.01) (Supplementary Figure 8B, and Supplementary Tables 1 and 2). The above data suggest that although the alternative culture conditions have minimal effects on the immunophenotypic profile of BMMSC and AdMSC, they significantly impact their differentiation potential.

### Alternative media formulations augment the proliferation capacity of BMMSC and AdMSC

**Figure 3:**
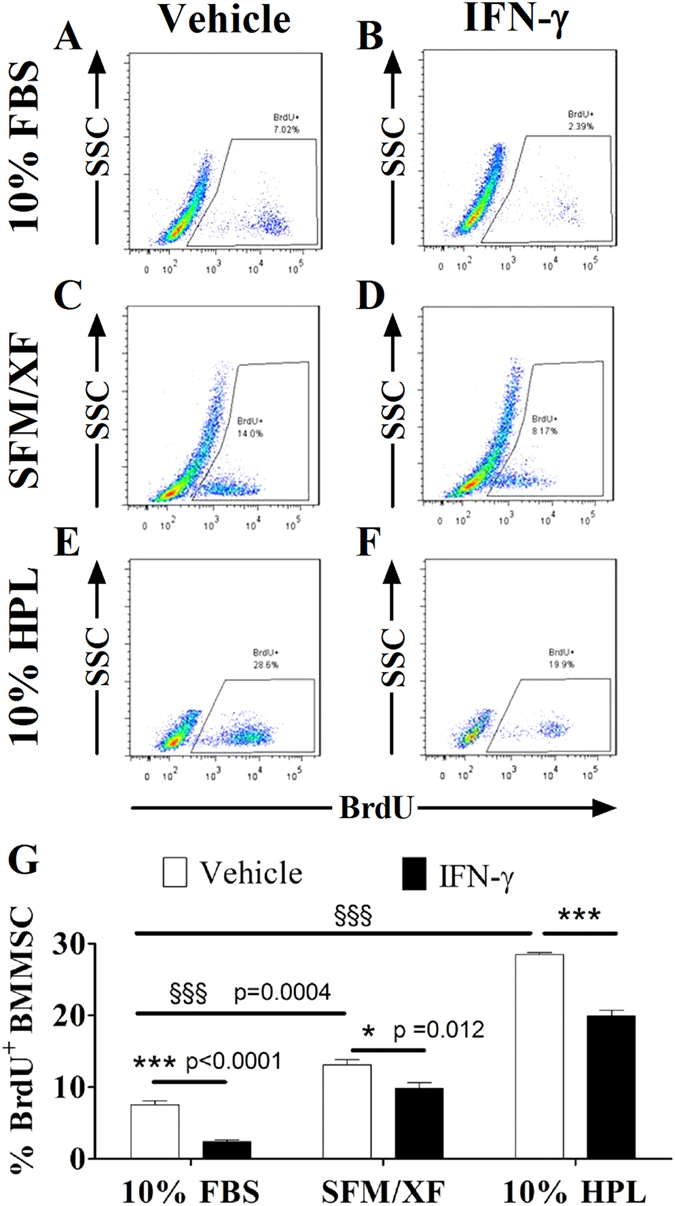
Effects of alternative culture media and IFN-γ priming on the proliferation capacity of BMMSC. Representative images of flow cytometric analysis of BrdU incorporation assays in resting and primed BMMSC expanded in 10%FBS (**A,B**), SFM/XF medium (**C,D**), 10%HPL (**E,F**). (**G**) Quantification of BrdU incorporation assays in BMMSC cells. All data points are generated by performing at least two independent experiments in triplicates. Data are mean values ± s.e.m.

**Figure 4:**
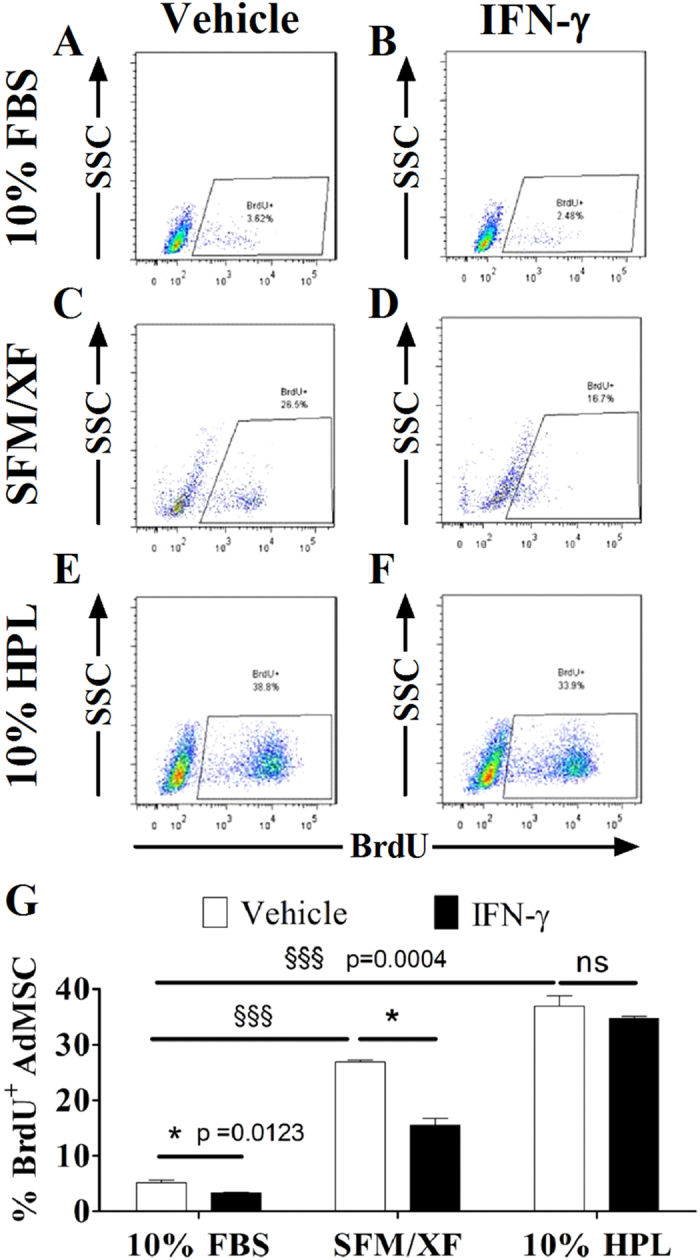
Effects of different media formulations and IFN-γ priming on the proliferation capacity of AdMSC. Representative images of flow cytometric analysis of BrdU incorporation assays in resting and primed AdMSC expanded in 10%FBS (**A,B**), SFM/XF medium (**C,D**), and 10%HPL (**E,F**). (**G**) Quantification of BrdU incorporation assays in AdMSC cells. All data points are generated by performing at least two independent experiments in triplicates. Data are mean values ± s.e.m.

Therapeutic application of MSC in inflammatory diseases, such as IBD, requires rapid *ex vivo* expansion of cells in conditions that do not compromise their immunomodulatory properties. Thus, we examined the effects of alternative media formulations on BMMSC and AdMSC proliferation via BrdU incorporation assays in resting and priming conditions. BrdU is a thymidine analog that incorporates into the DNA of the cell during the S-phase of the cell cycle. As shown in Fig. 3G (white bars), BMMSC-10%HPL followed by BMMSC-SFM/XF (p = 0.0004) exhibited significantly higher BrdU incorporation in resting conditions, compared with BMMSC-10%FBS (p = 0.0004). Similarly, AdMSC-10%HPL demonstrated the highest BrdU levels followed by AdMSC-SFM/XF, in comparison to AdMSC-10%FBS (p = 0.0004) (Fig. 4G, white bars). Representative images of BrdU flow cytometric analysis of BMMSC and AdMSC in all experimental conditions are presented in Figs 3A–F and 4A–F. IFN-γ priming significantly impaired the proliferation capacity of BMMSC in comparison to resting cells in all media formulations (BMMSC-10%FBS p < 0.0001, BMMSC-SFM/XF p = 0.012, and BMMSC-10%HPL p < 0.0001) (Fig. 3G, white vs black bars). Interestingly, IFN-γ priming decreased the proliferation capacity of AdMSC cultured in 10%FBS (p = 0.0123) and SFM/XF (p = 0.0123) but not in the 10%HPL (Fig. 4G, white vs black bars). Overall, the above results provide strong evidence that both alternative media formulations and particularly HPL-supplemented media can augment the growth potential of BMMSC and AdMSC. Additionally, we provide evidence that IFN-γ priming mediates anti-growth effects predominantly on BMMSC and to a lesser extent on AdMSC.

### Human platelet lysate compromises the immunosuppressive properties of BMMSC and AdMSC

**Figure 5:**
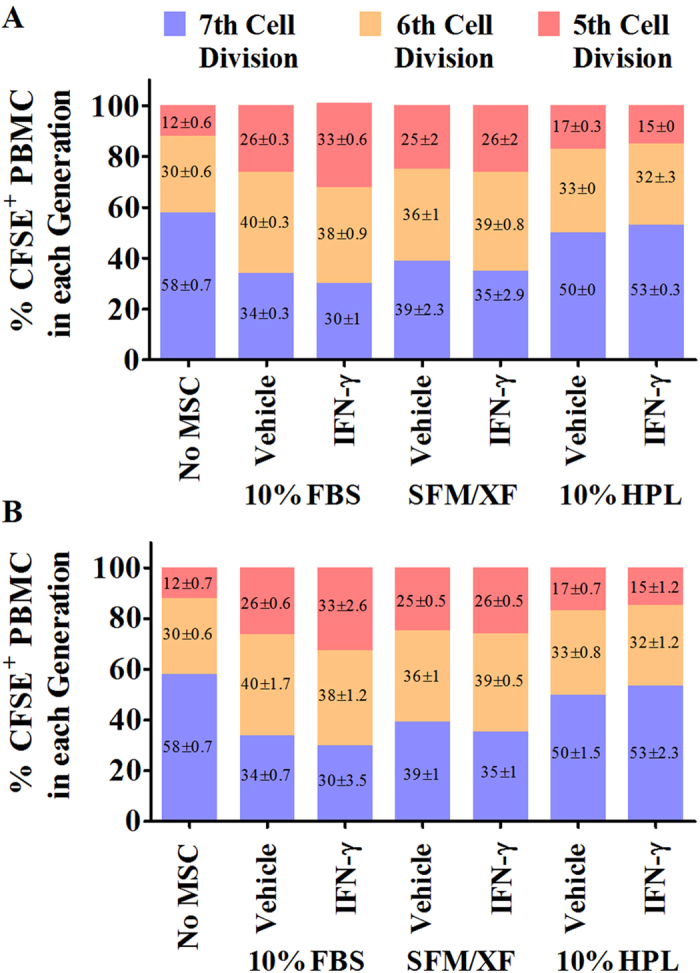
BMMSC and AdMSC expanded in FBS and SFM/XF, but not in HPL, inhibit the proliferation of PBMC. CFSE profiling of CD3^+^/CD28^+^ pre-stimulated PBMC co-cultured with resting and IFN-γ primed BMMSC (**A**) and AdMSC (**B**). Diagrams demonstrate the proportion of CFSE^+^ PBMC in the last three cell divisions in every condition. Pre-stimulated PBMC cultured in the absence of BMMSC were used as controls (No MSC). All data points are generated by performing at least two independent experiments in triplicates. Data are mean values ± s.e.m.

We assessed the immunosuppressive properties of BMMSC and AdMSC expanded in alternative media formulations in resting and primed conditions through co-culture assays of CFSE loaded human peripheral blood mononuclear cells (PBMC) with BMMSC and AdMSC. Resting BMMSC-10%FBS and AdMSC-10%FBS suppressed the proliferation of PBMC indicating an increase in their immunosuppressive properties. This effect was further enhanced (p < 0.05) by IFN-γ priming (Fig. 5A,B and Supplementary Tables 1 and 2). Similarly, resting BMMSC-SFM/XF decreased the proliferation capacity of PBMC (Fig. 5A,B). IFN-γ priming of BMMSC-SFM/XF did not further augment their immunosuppressive properties (Fig. 5A,B). On the contrary, BMMSC and AdMSC expanded in HPL-supplemented medium had no effect on the proliferation of PBMC in both resting and IFN-γ priming conditions (Fig. 5A,B). This result suggests that the immunosuppressive properties of BMMSC and AdMSC are inhibited by HPL-supplemented medium. Moreover, the pre-defined SFM/XF culture medium could represent an alternative to the FBS-supplemented medium as it maintains the immunosuppressive properties of BMMSC.

### BMMSC and AdMSC expanded in alternative media formulations show reduced levels of IDO-1

**Figure 6:**
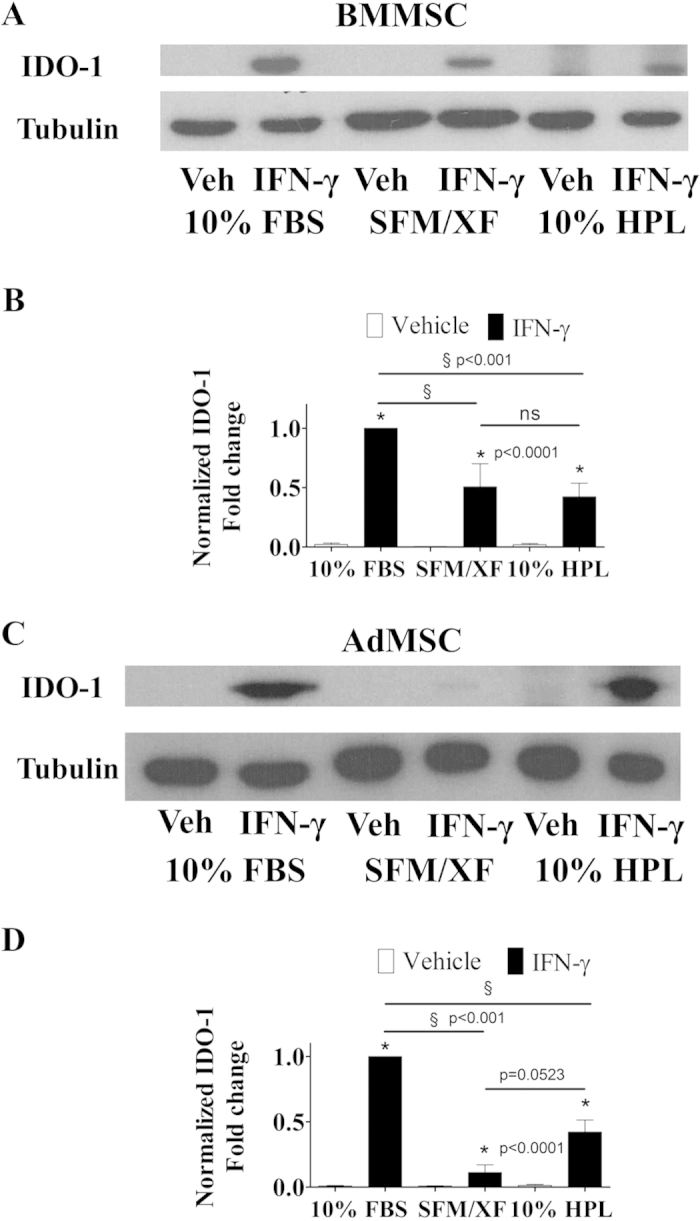
IFN-γ priming differentially regulates the expression of IDO-1 in BMMSC and AdMSC cultured in alternative media formulations. Western blot analysis of IDO-1 in (**A**) BMMSC and (**C**) AdMSC expanded in various culture media formulations. Quantification of IDO-1 expression levels in (**B**) BMMSC and (**D**) AdMSC. All data points are generated by performing at least two independent experiments in triplicates. Data are mean values ± s.e.m. * indicate comparison between Vehicle and IFN-γ at every condition.

Indoleamine 2, 3-dioxygenase 1 (IDO-1) is an established mediator of the immunosuppressive properties of human MSC. To examine the levels of IDO-1, we performed protein expression analysis in BMMSC and AdMSC expanded in all media formulations. Under resting conditions IDO-1 was not detected in BMMSC and AdMSC expanded in all media formulations (Fig. 6A–D, respectively). However, following IFN-γ priming IDO-1 was significantly (p < 0.0001) up-regulated (Fig. 6A–D, respectively). IFN-γ treated BMMSC-10%FBS expressed the highest levels of IDO-1 followed by BMMSC-SFM/XF and BMMSC-10%HPL (p < 0.001) (Fig. 6A,B). Similarly, IFN-γ primed AdMSC-FBS expressed the highest levels of IDO-1 followed by AdMSC-10%HPL and AdMSC-SFM/XF (p < 0.001) (Fig. 6C,D). Hence, both SFM/XF and HPL-supplemented media increase IDO-1 expression following IFN-γ treatment albeit to a lesser extent than 10%FBS.

Analytical tables summarizing all results obtained from non-primed and IFN-γ primed BMMSC and AdMSC expanded in all media-formulations are presented in Supplementary Table 1 and Table 2.

## Discussion

MSC offer new therapeutic strategies for the treatment of refractory cases of IBD and other inflammatory diseases. Administering therapeutically meaningful numbers of MSC in patients requires extensive *in vitro* cell propagation utilizing FBS as the main source of growth supplement. However, various side effects related to the utilization of animal derived products such as FBS for the development of MSC have been reported. Thus, there is an unmet need for the identification of novel animal free culture conditions for the expansion of MSC. HPL can be obtained from volunteer blood donors and is available in most hospitals and transfusion centers. Since HPL is produced under institutional standard operating procedures, and per AABB (American Association of Blood Banks) requirements and U.S. Food and Drug Administration (FDA) regulations, it is a safe alternative medium for MSC production. HPL can sustain the growth of MSC without affecting their immunophenotype. On the other hand, recent reports have indicated that HPL can either maintain or diminish the immunosuppressive properties of MSC^27^. Thus the effects of HPL on the immunosuppressive properties of MSC remain controversial and require further investigation.

The serum-free/xeno-free medium (SFM/XF) is a commercially available product that meets good manufacturing practice (GMP) standards and was recently approved by the (FDA) for expansion of MSC towards therapeutic applications^42^,^45^. Still, very little is known about the effects of SFM/XF on the immunosuppressive properties of BMMSC and AdMSC. Therefore, the goal of the current study is to perform a comprehensive comparison of the functional properties of resting and IFN-γ primed BMMSC and AdMSC expanded in alternative culture media formulations (HPL and SFM/XF).

We found that both alternative media formulations (HPL and SFM/XF) significantly enhanced the proliferation capacity of MSC as evidenced by the increased BrdU incorporation rate. These results are in agreement with previous studies demonstrating that HPL-supplement and SFM/XF can augment the proliferation of BMMSC and AdMSC^35^,^45^. Substitution of FBS by HPL or SFM/XF can reduce the required amount of MSC manufacturing time and accelerate the production of MSC in therapeutically relevant numbers.

Additionally, our data shows that IFN-γ priming mediates anti-proliferative effects on both BMMSC and AdMSC. However, IFN-γ primed BMMSC and AdMSC demonstrate superior immunosuppressive properties and thus represent potential potent candidates for future clinical trials of immunological disorders. Further exploration of their function under serum-free expansion conditions is needed. The effects of alternative media formulations on IFN-γ primed MSC have been largely overlooked. This is of paramount importance for the design of future MSC clinical trials, as priming with pro-inflammatory cytokines dramatically increases their anti-inflammatory properties.

Our data also suggests that expansion of both BMMSC and AdMSC in HPL supplemented media abolishes their immunosuppressive properties. BMMSC-10%HPL and AdMSC-10%HPL were unable to block the proliferation of CD3^+^/CD28^+^ activated T-cells in comparison to BMMSC-10%FBS and AdMSC-10%FBS. Priming of both cell types with IFN-γ did not increase their immunosuppressive properties in contrast to the respective cell types expanded in FBS. In a recent paper by Menard *et al.* it was shown that BMMSC and AdMSC expanded in HPL-supplemented media maintained their immunosuppressive properties similarly to BMMSC expanded in FBS^49^. The observed discrepancies between the current manuscript and the one by Menard *et al.* are most likely attributed to differences in the HPL production (apheresis-procedure versus whole blood processing). Previous studies have shown that whole blood derived HPL and apheresis derived HPL have similar growth promoting capacities on MSC^33^. Our work suggests that although apheresis derived HPL is a potent growth promoting supplement, it abrogated the immunosuppressive properties of MSC. Overall, HPL needs to be carefully evaluated as an alternative growth medium for MSC due to the documented discrepancies on the immunosuppressive properties of the cells.

SFM/XF represents the first FDA-approved xeno-free culture media for the expansion of BMMSC and AdMSC towards clinical applications. SFM/XF is a GMP-compatible serum-free/xeno-free culture medium with a trademarked list of components, including but not limited to human albumin, lipoproteins, and recombinant human growth factors^44^. Our data show that SFM/XF not only augments the proliferation capacity of BMMSC and AdMSC but preserves their immunosuppressive properties. Interestingly, IFN-γ priming of the SFM/XF-expanded BMMSC and AdMSC cells did not further potentiate their immunosuppressive properties.

Our study offers a detailed characterization of MSC expanded in various media formulations that can serve as a foundation for the development of cell therapy protocols. Our data provides a strong framework for the development of a serum-free/xeno-free GMP-compatible manufacturing practice for the expansion of BMMSC and AdMSC.

## Methods

### MSC expansion

Human donor derived BMMSC and AdMSC were purchased from Lonza (Basel, Switzerland). BMMSC and AdMSC were initially expanded in FBS-supplemented Dulbecco’s modified Eagle’s-low glucose medium (DMEM, Invitrogen, Carlsbad, CA, USA) and were frozen at passage 1 to serve as a master cell bank. More specifically, BMMSC and AdMSC were grown in 10 cm^2^ non-coated plastic vessels (Corning, Corning, NY, USA) in a 37 °C humidified incubator containing 5% CO_2_. When cells reached 80% confluence they were trypsinized (0.05% Trypsin/EDTA, Invitrogen) and were subsequently re-seeded at a density of 4,000 cells per cm^2^ in alternative media formulations. The remainder of the cells were frozen in 90%FBS (Hyclone, Bonn, Germany) and 10% dimethyl sulfoxide (DMSO, Sigma, Aldrich, St Louis, MO, USA).

Alternative media formulations consisted of DMEM supplemented with 10%HPL (BMMSC-10%HPL, and AdMSC-10%HPL) and a commercially available serum-free/xeno-free culture medium (BMMSC-SFM/XF, AdMSC-SFM/XF) (StemPro® MSC SFM XenoFree termed SFM/XF, Invitrogen). HPL-supplemented medium contained heparin (2 IU/mL, Sigma), 1% penicillin/streptomycin (Gibco, Garlsbad, CA, USA), and 2 mM Glutamax (Gibco). SFM/XF culture medium was also supplemented with Glutamax (2 mM) according to manufacturer’s instructions. BMMSC-SFM/XF and AdMSC-SFM/XF were grown on CELLstart™ xeno-free substrate (Invitrogen) according to manufacturer’s instructions. All experiments were performed in comparison to BMMSC and AdMSC expanded in basal DMEM-medium supplemented with 10%FBS and 1% penicillin/streptomycin (BMMSC-10%FBS, AdMSC-10%FBS).

### MSC priming

To generate IFN-γ pre-stimulated (primed) BMMSC and AdMSC, cells were seeded at the same density in all media formulations supplemented with 50 ng/mL of recombinant human IFN-γ (Sigma). IFN-γ treatment lasted three days as previously described^14^. All experiments were performed in comparison to vehicle- (1× phosphate buffer solution, PBS, supplemented with 0.1% bovine serum albumin, BSA) treated cells.

### MSC cell size analysis

Cell size (area) of all MSC expanded in various media formulations was measured by a blinded investigator using Image J. All images were taken using an EVOS XL Core Cell Imaging System under 20× magnification objective. The cell size of the following number of cells was counted from each experimental condition: BMMSC-10%FBS (Vehicle; n = 57, IFN-γ; n = 42), BMMSC-SFM/XF (Vehicle; n = 65, IFN-γ; n = 39), BMMSC-10%HPL (Vehicle; n = 51, IFN-γ; n = 50), AdMSC-10%FBS (Vehicle; n = 60, IFN-γ; n = 50), AdMSC-SFM/XF (Vehicle; n = 60, IFN-γ; n = 83), and AdMSC-10%HPL (Vehicle; n = 65, IFN-γ; n = 56). Images were obtained from multiple (5–6) optical fields of each cell culture plate.

#### Human platelet lysates (HPL) preparation

HPL was manufactured via three consecutive freeze and thaw cycles^40^ of platelet lysates obtained from the single donor apheresis platelet unit collected by Trima Accel cell separator (Trima, Terumo BCT) at the UCLA Blood and Platelet Center; these units failed to meet minimum platelet yield requirements and were not suitable for transfusion. Platelet rich plasma (PRP) prepared by cytapheresis was obtained from a clinical transfusion laboratory in UCLA. All samples were tested negative for common serology prior to usage. To avoid platelet clotting, all samples were supplemented with anti-coagulant (Heparin). PRP derived from five blood donors were combined by sterile connection to obtain a single homogenous product. The PRP used in the following studies was prepared from separate donors of blood group O. Subsequently, PRP was aliquoted in sterile tubes (50 mL) under aseptic conditions and aliquots underwent three cycles of freeze and thaw to promote efficient release of growth factors. To remove cellular debris, all processed samples were centrifuged at 4,000 g at 4 °C for 15 minutes. Supernatant was transferred to new sterile tubes and frozen at −80 °C.

### MSC immunophenotypic analysis

Immunophenotypic analysis of expanded MSC was carried out using flow cytometry for the following markers: CD73, CD90, CD14, CD34, CD45, CD105, and MHCII (HLA-DR) (BD Biosciences)^48^. Samples were analyzed using a LSRII Flow Cytometer (BD Biosciences, San Jose, CA, USA) and the obtained data were analyzed with FlowJo software (FlowJo, version 7.6.1).

#### BrdU assays

BMMSC-10%FBS, BMMSC-SFM/XF, BMMSC-10%HPL, AdMSC-10%FBS, AdMSC-SFM/XF, and AdMSC-10%HPL were seeded at a density of 2000 cells/cm^2^ and 24 hours later were treated with IFN-γ (50 ng/mL) for a period of 72 hours. At day 3, BrdU (10 μM, BD Biosciences) was added in the culture medium and was allowed to incorporate into the DNA of the cells for three hours prior to flow cytometric analysis.

#### CFSE assays

BMMSC and AdMSC were primed with IFN-γ (50 ng/mL) for a period of 3 days prior to their seeding into a 96-well plate. One day after IFN-γ priming, human peripheral blood mononuclear cells (PBMC), isolated from a single healthy donor, were stimulated with CD3^+^/CD28^+^ beads (Invitrogen) for a period of 3 days. CD3^+^/CD28^+^ -stimulated PBMC were pre-loaded with carboxyfluorescein succinimidyl ester (CFSE, Invitrogen) fluorescent dye and were cultured with primed BMMSC and AdMSC in various PBMC/MSC cell number ratios. CFSE is commonly used to monitor lymphocyte proliferation due to its progressive equal sharing within daughter cells following each cell division. CFSE fluorescence was assessed 96 hours following co-culture of MSC and PBMC via flow cytometric analysis.

#### Immunoblot analysis

BMMSC and AdMSC were washed with ice-cold PBS and incubated with assay buffer containing dithiothreitol (DTT) and protease inhibitors phenylmethylsulfonyl fluoride and sodium orthovanadate. The insoluble debris was removed by centrifugation (14,000 rpm, 5 min, and 4 °C) and supernatants were analyzed by immunoblot analysis. Equal amount of cell lysates (20–30 μg) were loaded and transferred to a nitrocellulose membrane. The membrane was blocked with 1X Tris-Buffered Saline (TBS) containing 0.1% Tween-20 and 10% milk. The membranes were incubated with primary antibodies against IDO-1 and Tubulin-α (Cell Signaling, Boston, MA, USA) overnight at 4 °C under constant shaking. Appropriate secondary pexodisase antibodies (goat-anti-rabbit IgG and goat-anti-mouse IgG, Sigma) were applied for 2 hours at ambient temperature. Membranes were washed with 1X TBS-T containing 0.1% Tween-20. Enhanced chemiluminescence (ECL) was applied for the detection of horseradish peroxidase (HRP) activity from protein probes.

### MSC differentiation assays

MSCs were seeded in 12-well culture plates at a density of 2500 cells/cm^2^ and expanded until cells reached 80–90% confluency. For osteogenic and adipogenic differentiation and immuno-staining experiments, cells were stimulated for 14 days in differentiation media (GIBCO) according to manufacturer’s instructions. In the experiments intended for quantification of the differentiation potential, MSC were cultured in differentiation media for 7 days. Osteogenic and adipogenic differentiation were assessed by staining for alkaline phosphatase (Fast Blue, Sigma) and for the presence of lipid droplets (Oil red O, Sigma)^14^. Quantitative real time polymerase chain reaction (qRT-PCR) was utilized for quantification of osteogenic and adipogenic differentiation efficacy in the various alternative media formulations. To quantify the effect of alternative media on the adipogenic and osteogenic differentiation, the expression of adiponectin, PPAR-γ, and alkanine phosphatase was assessed using the following sets of primers: PPAR-γ; Forward: GAGCCCAAGTTTGAGTTTGC, Reverse: TCAATGGGCTTCACATTCAG, Adiponectin; Forward: CCTGGTGAGAAGGGTGAGAA, Reverse: CTCCTTTCCTGCCTTGGATT, Alkaline phosphatase: Forward: GACATCGCCTACCAGCTCAT, Reverse: TGGCTTTCTCGTCACTCTCA.

#### Statistical Analysis

Statistical differences between groups were evaluated using one way Anova analysis and Student’s unpaired t-test, using GraphPad Prism (Version 5.03). All data are presented as mean ± SE. A p-value ≤ 0.05 was considered statistically significant.

## Additional Information

**How to cite this article**: Oikonomopoulos, A. *et al.* Optimization of human mesenchymal stem cell manufacturing: the effects of animal/xeno-free media. *Sci. Rep.* **5**, 16570; doi: 10.1038/srep16570 (2015).

## Supplementary Material

Supplementary Information
