# Supplementary material for: Optimization of human mesenchymal stem cell manufacturing: the effects of animal/xeno-free media
Source: Sci Rep. 2015 Nov 13;5:16570. doi: 10.1038/srep16570 (PMC4643287; doi:10.1038/srep16570)
Supplement: Supplementary Information [file srep16570-s1.pdf]

Title:

Optimization of human mesenchymal stem cell manufacturing: the effects of animal/xeno-free media

Author list:

Angelos Oikonomopoulos[1]*, Welmoed K. van Deen[1,2], Aida-Rae Manansala[3], Precious N. Lacey[1], Tamera A. Tomakili[1], Alyssa Ziman[3], Daniel W. Hommes[1]

Affiliations:

[1]Center for Inflammatory Bowel Diseases, Melvin and Bren Simon Digestive Diseases Center, David Geffen School of Medicine, UCLA, Los Angeles, California.

[2]Department of Gastroenterology and Hepatology, Leiden University Medical Center, Leiden, The Netherlands.

[3]Division of Transfusion Medicine, Department of Pathology and Laboratory Medicine, David Geffen School of Medicine, UCLA, Los Angeles, California.

Supplemental Figure 1

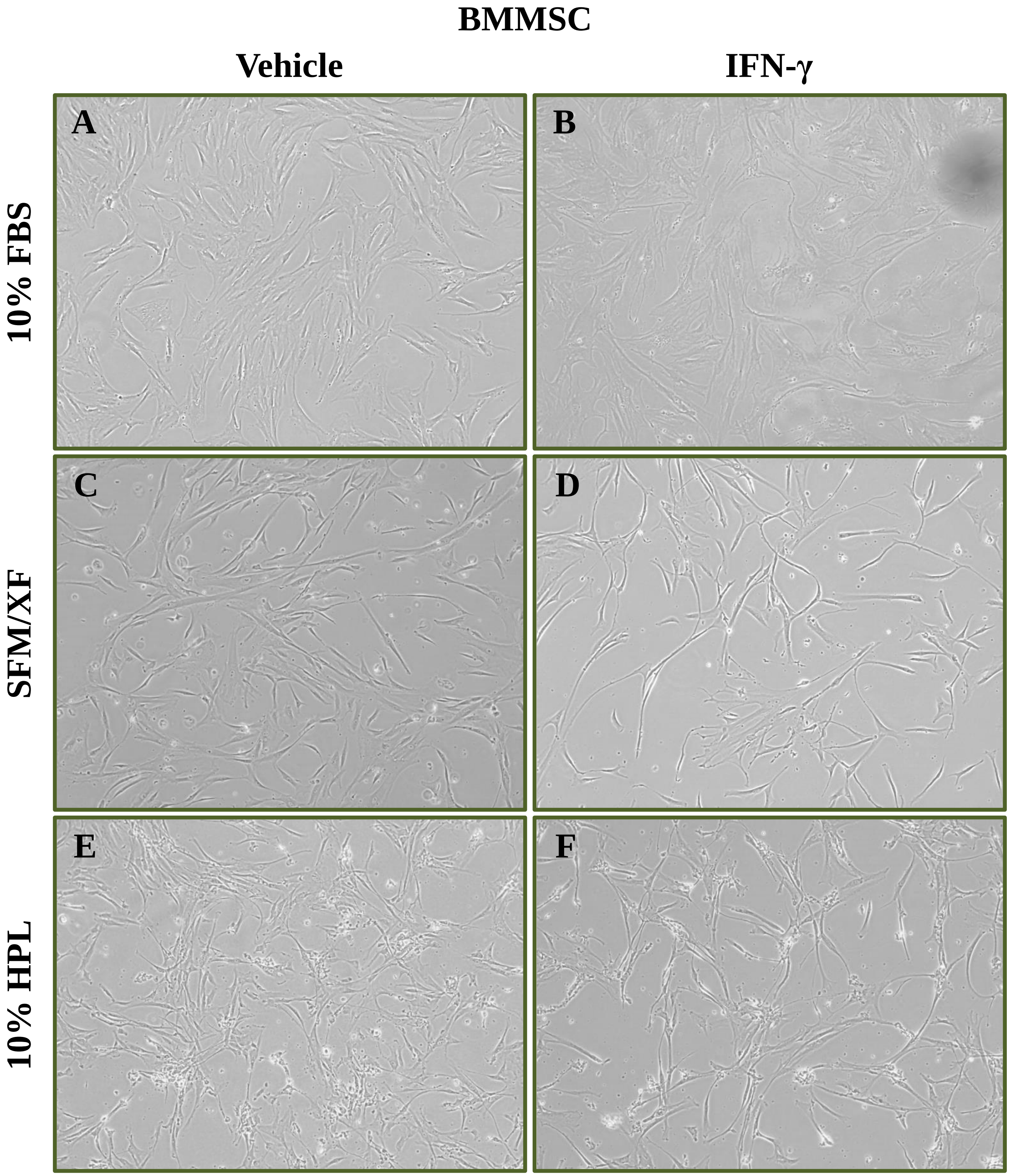

Supplemental Figure 2

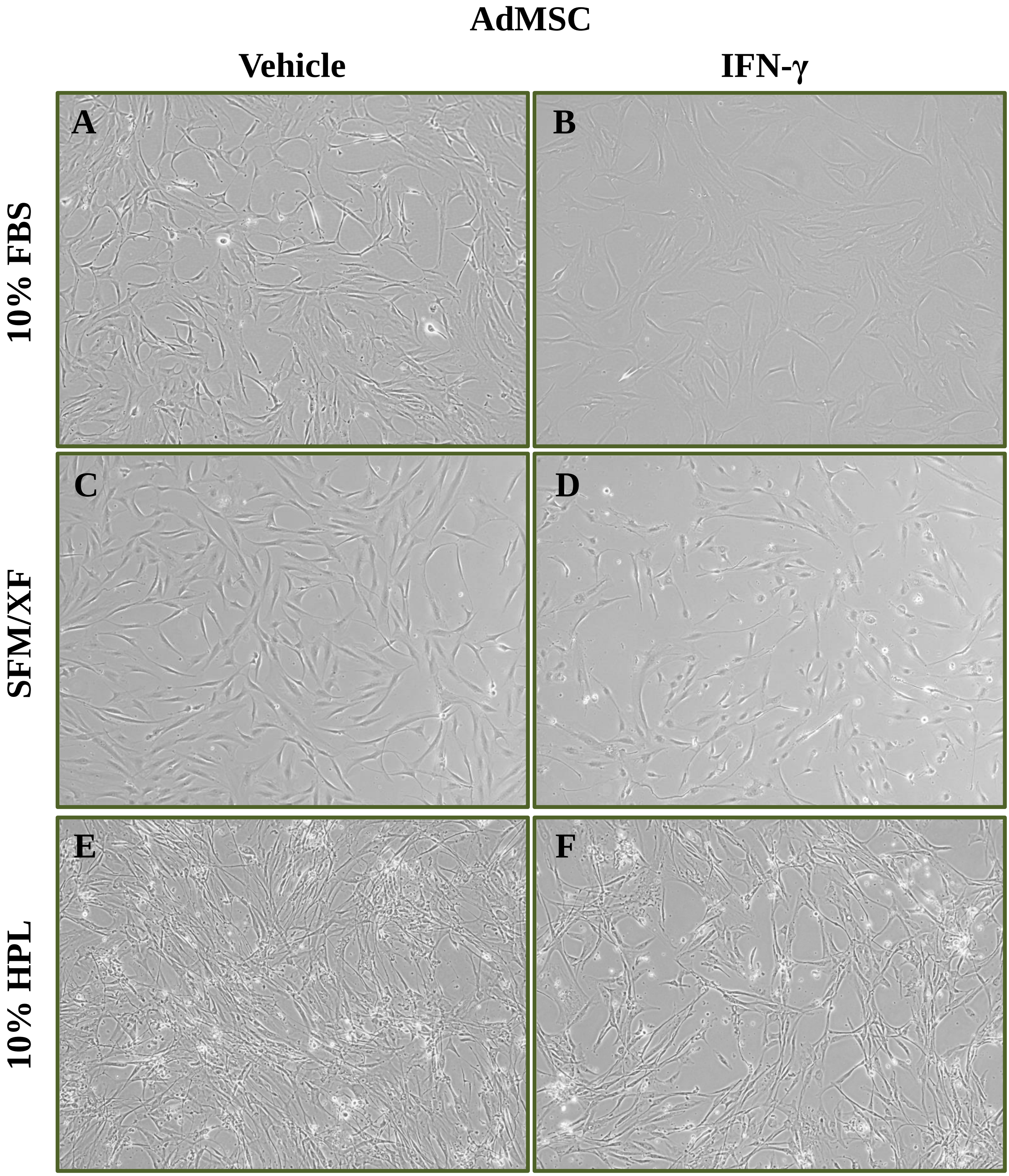

Supplemental Figure 3

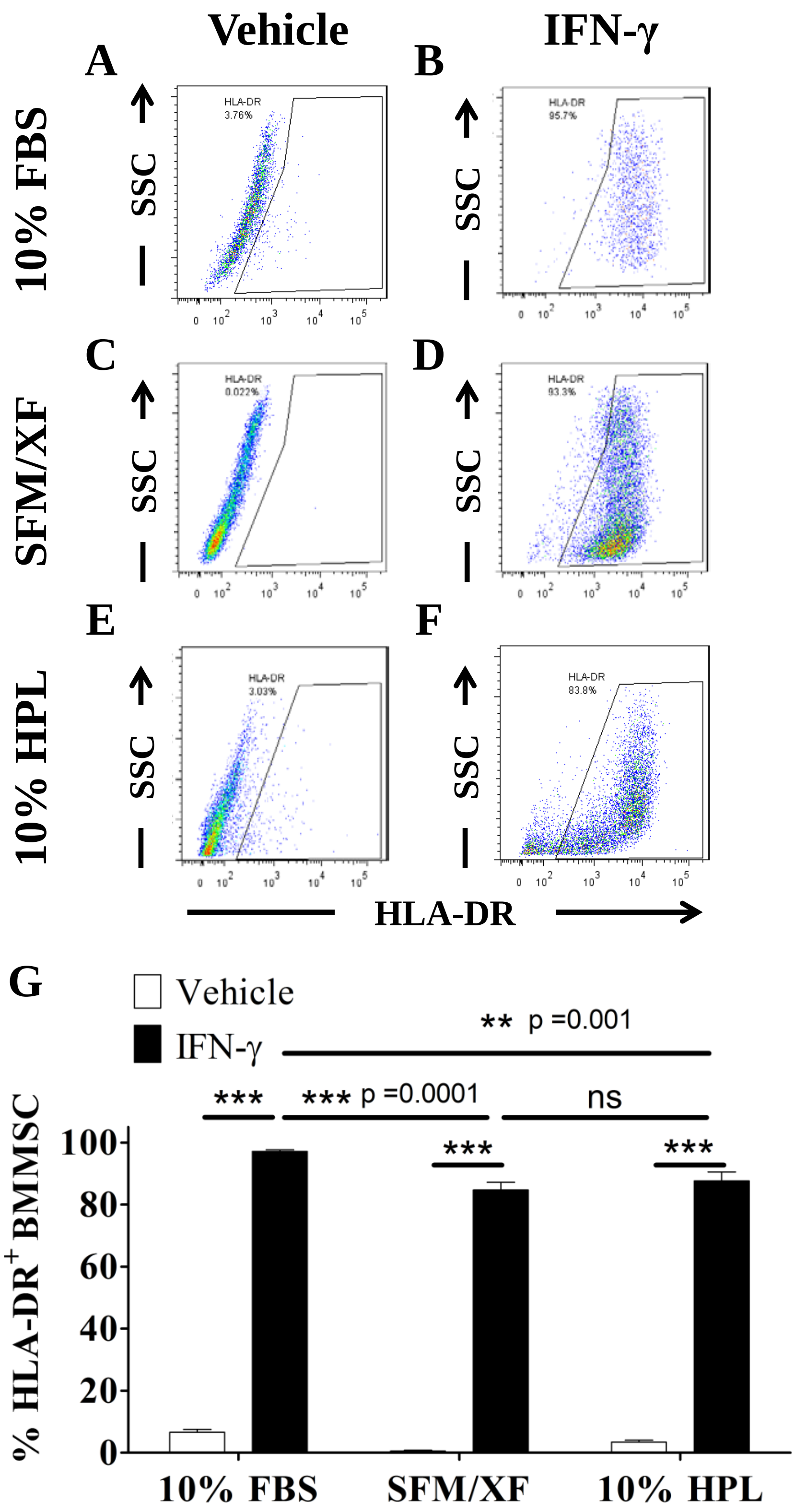

Supplemental Figure 4

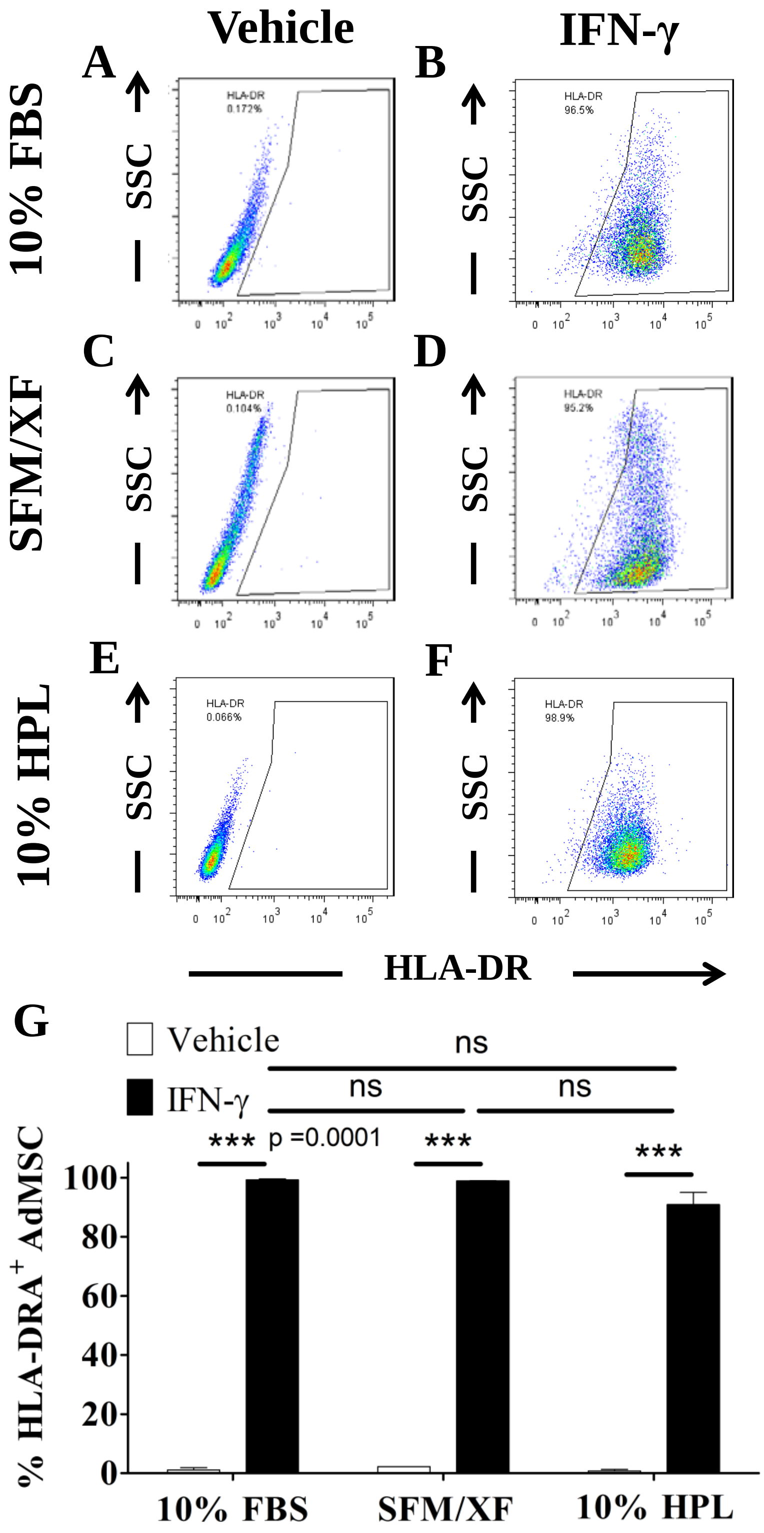

Supplemental Figure 5

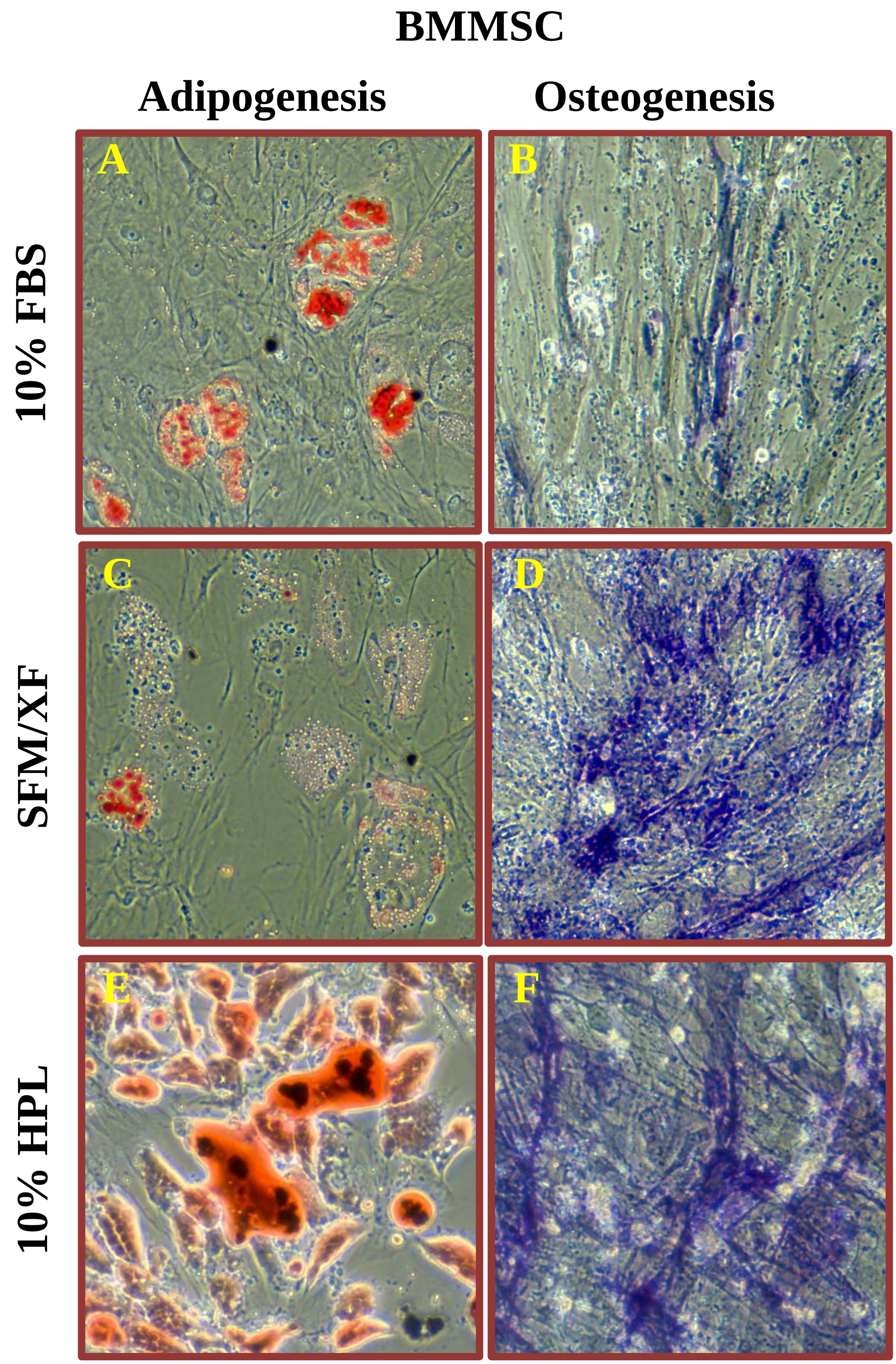

Supplemental Figure 6

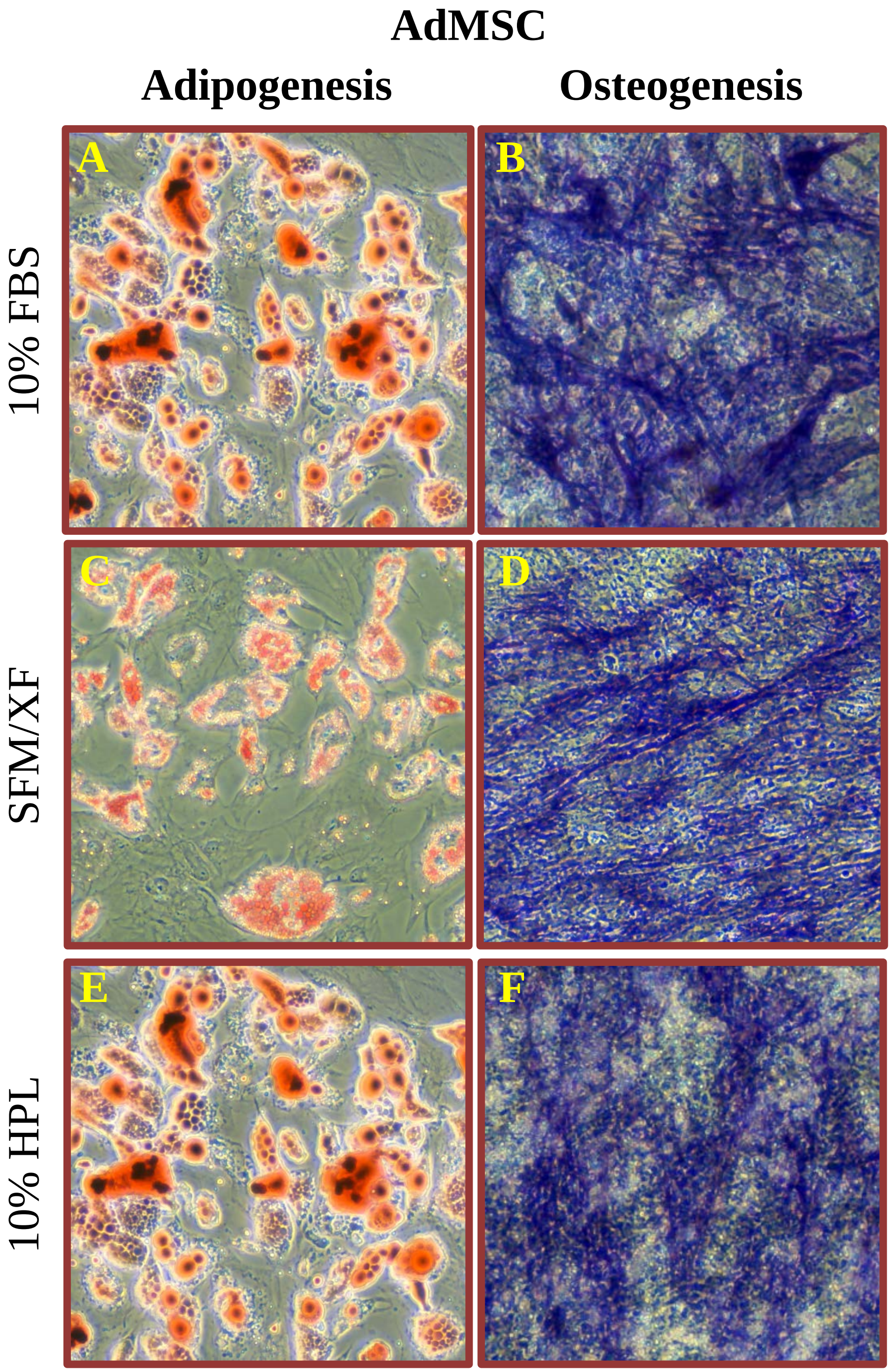

Supplemental Figure 7

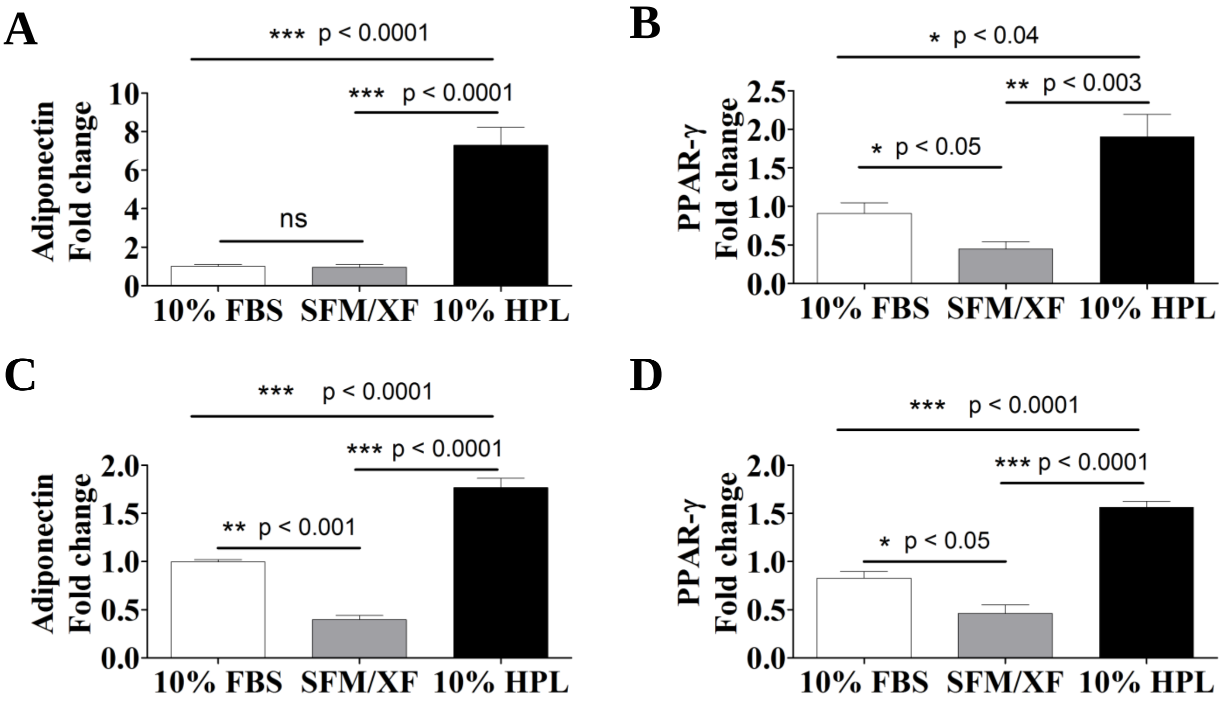

Supplemental Figure 8

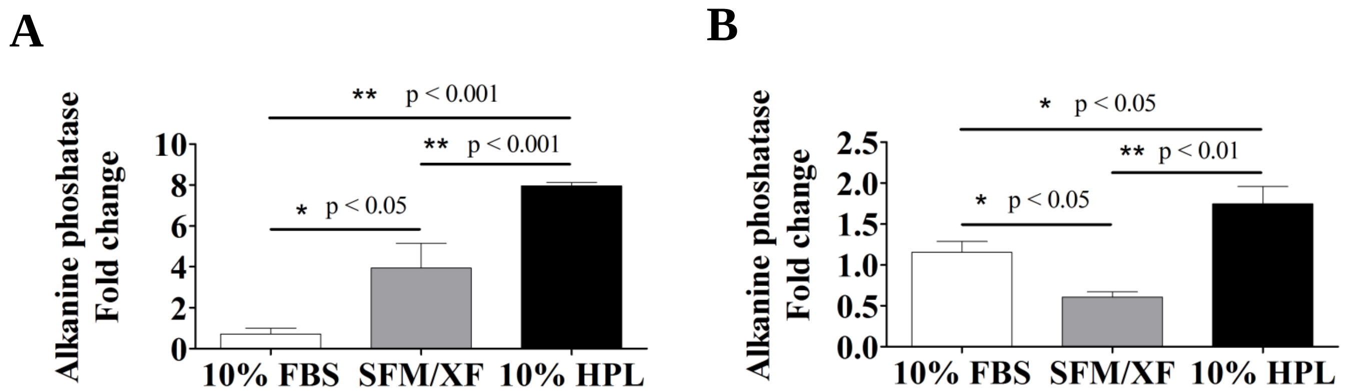

Supplemental Tables

Supplemental Table 1

Cell type	Media formulation	Cell size	Markers	HLA-DR (primed MSC)	Adipogenesis	Osteogenesis	BrdU	Immunomodulation (CFSE assay)	IDO-1
BMMSC	SFM/XF compared to 10% FBS	—	—	↓	—	↑↑	↑	↓	↓
BMMSC	10% HPL compared to 10% FBS	↓	—	↓	↑↑↑	↑↑↑	↑↑↑	↓↓↓	↓↓
AdMSC	SFM/XF compared to 10% FBS	↓	—	—	↓	↓	↑↑	↓	↓↓↓
AdMSC	10% HPL compared to 10% FBS	↓	—	—	↓	↑↑	↑↑↑	↓↓↓	↓↓

Supplemental Table 2

Cell type	Media formulation	Treatment compared to vehicle	Cell size	Markers	HLA-DR	BrdU	Immunomodulation (CFSE assay)	IDO-1
BMMSC	10% FBS	IFN-γ	↑	—	↑↑	↓↓	↑↑↑	↑↑↑
BMMSC	SFM/XF	IFN-γ	—	—	↑↑	↓	↑↑	↑↑
BMMSC	10% HPL	IFN-γ	—	—	↑↑	↓↓	↑↑	↑↑
AdMSC	10% FBS	IFN-γ	↑↑	—	↑↑	↓	↑↑↑	↑↑↑
AdMSC	SFM/XF	IFN-γ	↑	—	↑↑	↓↓	↑	↑
AdMSC	10% HPL	IFN-γ	—	—	↑↑	—	↑↑	↑↑

Supplemental Material

Supplemental Figure 1: Representative bright-field images of BMMSC expanded in 10% FBS (A-B), SFM/XF medium (C-D), and 10% HPL (E-F).

Supplemental Figure 2: Representative bright-field images of AdMSC cultured in 10% FBS (A-B), SFM/XF medium (C-D), and 10% HPL (E-F).

Supplemental Figure 3: IFN-γ priming stimulates the expression of HLA-DR in BMMSC in all media formulations. Representative images of flow cytometric analysis of HLA-DR expression in resting and primed BMMSC expanded in 10% FBS (A-B), SFM/XF (C-D), 10% HPL (E-F). (G) Quantification of HLA-DR expression analysis. All data points are generated by performing at least two independent experiments in triplicates. Data are mean values ± s.e.m.

Supplemental Figure 4: IFN-γ priming stimulates the expression of HLA-DR in AdMSC in all media formulations. Representative images of flow cytometric analysis of HLA-DR expression in resting and primed AdMSC expanded in 10% FBS (A-B), SFM/XF (C-D), 10% HPL (E-F). (G) Quantification of HLA-DR expression analysis. All data points are generated by performing at least two independent experiments in triplicates. Data are mean values ± s.e.m.

Supplemental Figure 5: BMMSC retain differentiation potential when cultured in alternative media formulations. Representative images of adipogenic (A, C, E) and osteogenic (B, D, F) differentiation of BMMSC expanded in 10% FBS (A-B), SFM/XF (C-D), and 10%

HPL (E-F). Red and blue colors indicate the presence of lipid droplets (A, C, E) and expression of alkaline phosphatase (B, D, F) respectively.

Supplemental Figure 6: AdMSC retain differentiation potential when cultured in alternative media formulations. Representative images of adipogenic (A, C, E) and osteogenic (B, D, F) differentiation of AdMSC expanded in 10% FBS (A-B), SFM/XF (C-D), and 10% HPL (E-F). Red and blue colors indicate the presence of lipid droplets (A, C, E) and expression of alkaline phosphatase (B, D, F) respectively.

Supplemental Figure 7: BMMSC and AdMSC expanded in HPL demonstrate higher adipogenic differentiation capacity. Quantitative real-time PCR analysis (qRT-PCR) of adiponectin and PPAR-γ in BMMSC and AdMSC expanded in all media formulations. BMMSC-10%HPL (A-B) and AdMSC-10%HPL (C-D) expressed higher levels of adiponectin and PPAR-γ, followed by MSC expanded in 10%FBS and SFM/XF. All data points are generated by performing at least two independent experiments in triplicates. Data are mean values ± s.e.m.

Supplemental Figure 8: Alternative media formulations affect BMMSC and AdMSC differentiation capacity. Quantitative real-time PCR analysis (qRT-PCR) of alkaline phosphatase in BMMSC and AdMSC expanded in all media formulations. BMMSC-10%HPL (A) and AdMSC-10%HPL (B) expressed higher levels of alkaline phosphatase compared to all other cell types. BMMSC-SFM/XF and AdMSC-10%FBS expressed higher levels of alkaline phosphatase in comparison to BMMSC-10%FBS and AdMSC-SFM/XF respectively. All data points are generated by performing at least two independent experiments in triplicates. Data are mean values ± s.e.m.

Supplemental Table 1: Summary of the effects of alternative media formulations on the various properties of BMMSC and AdMSC. All properties have been compared to BMMSC and AdMSC expanded in 10% FBS. (-) No effect, (↑) increased, and (↓) decreased effect, between compared cell pairs.

Supplemental Table 2: Summary of the effects of IFN-γ on BMMSC and AdMSC expanded in all alternative media formulations. All comparisons have been made to vehicle-treated MSC. (-) No effect, (↑) increased, and (↓) decreased effect, between compared cell pairs.
